# How to evaluate perfusion imaging in post-treatment glioma: a comparison of three different analysis methods

**DOI:** 10.1007/s00234-024-03374-3

**Published:** 2024-05-08

**Authors:** Siem D. A. Herings, Rik van den Elshout, Rebecca de Wit, Manoj Mannil, Cécile Ravesloot, Tom W. J. Scheenen, Anne Arens, Anja van der Kolk, Frederick J. A. Meijer, Dylan J. H. A. Henssen

**Affiliations:** 1 Department of Medical Imaging, Radboud University Medical Center, Nijmegen, The Netherlands; 2 Radboudumc Center of Expertise Neuro-Oncology, Nijmegen, The Netherlands; 3 University Clinic for Radiology, Westfälische Wilhelms-University Muenster and University Hospital Muenster, Albert-Schweitzer-Campus 1, E48149 Muenster, Germany

**Keywords:** Volume of interest, Dynamic susceptibility contrast, Perfusion weighted magnetic resonance imaging, Glioma, Tumor progression, Treatment-related abnormality

## Abstract

**Introduction:**

Dynamic susceptibility contrast (DSC) perfusion weighted (PW)-MRI can aid in differentiating treatment related abnormalities (TRA) from tumor progression (TP) in post-treatment glioma patients. Common methods, like the ‘hot spot’, or visual approach suffer from oversimplification and subjectivity. Using perfusion of the complete lesion potentially offers an objective and accurate alternative. This study aims to compare the diagnostic value and assess the subjectivity of these techniques.

**Methods:**

50 Glioma patients with enhancing lesions post-surgery and chemo-radiotherapy were retrospectively included. Outcome was determined by clinical/radiological follow-up or biopsy. Imaging analysis used the ‘hot spot’, volume of interest (VOI) and visual approach. Diagnostic accuracy was compared using receiving operator characteristics (ROC) curves for the VOI and ‘hot spot’ approach, visual assessment was analysed with contingency tables. Inter-operator agreement was determined with Cohens kappa and intra-class coefficient (ICC).

**Results:**

29 Patients suffered from TP, 21 had TRA. The visual assessment showed poor to substantial inter-operator agreement (κ = -0.72 – 0.68). Reliability of the ‘hot spot’ placement was excellent (ICC = 0.89), while reference placement was variable (ICC = 0.54). The area under the ROC (AUROC) of the mean- and maximum relative cerebral blood volume (rCBV) (VOI-analysis) were 0.82 and 0.72, while the rCBV-ratio (‘hot spot’ analysis) was 0.69. The VOI-analysis had a more balanced sensitivity and specificity compared to visual assessment.

**Conclusions:**

VOI analysis of DSC PW-MRI data holds greater diagnostic accuracy in single-moment differentiation of TP and TRA than ‘hot spot’ or visual analysis. This study underlines the subjectivity of visual placement and assessment.

## Introduction

In the post-therapeutic setting of gliomas, it has been recommended to perform MRI within two days after surgery to assess the extent of resection, the presence of residual tumor tissue and the detection of complications [1]. However, an increasingly important part of neuroimaging concerns the visualisation of biological changes and the (early) prediction of response during or after treatment. In the follow-up imaging of gliomas treatment decisions are based on the observed effects, or lack of effect of the treatment. The Response Assessment in Neuro-Oncology (RANO) guidelines state that the response to therapy can be assessed by studying the extent of the T2/FLAIR signal and the size of the post-contrast enhancing region on the T_1_-weighted MRI over time [2]. However, early detection of tumor progression (TP) in post-treatment imaging of glioma is complicated due to the occurrence of treatment-related abnormalities (TRA; e.g., pseudoprogression and radiation necrosis). TRA and TP have similar imaging features on conventional MRI sequences, which results in poor differentiation between these two entities [3]. Therefore, more advanced MRI techniques (e.g., diffusion weighted and perfusion weighted MRI) play an important role in this setting.

Perfusion weighted imaging (PWI) is sensitive to changes in hemodynamics by differences in (micro-)vascularization such as local (neo)vascularization; it is therefore widely implemented in glioma imaging [4]. Three main PWI techniques are known: dynamic susceptibility contrast (DSC) imaging, dynamic contrast enhancing (DCE) imaging and arterial spin labelling (ASL). In ASL, labelling of bloodflow is used as endogenous contrast to measure perfusion, whereas the other two techniques use exogenous, gadolinium-based contrast agents which are intravenously injected [5]. DSC imaging is most commonly used in glioma imaging[4], as it is the most accurate and sensitive of the mentioned perfusion techniques [6], while also having a short acquisition time [7]. DSC perfusion imaging is based on a T_2_*-weighted pulse sequence in which the susceptibility induced signal loss resulting from the passage of a bolus of contrast agent is used for perfusion analysis. Various meta-analyses underline the diagnostic capacity of DSC perfusion-weighted imaging in the post-treatment setting to distinguish TRA from TP [8–10]. Despite its promise, perfusion–derived biomarkers have yet to be widely adopted. This discrepancy in potential and actual usage can be attributed to a shortfall of clinical technical skill, time constraints [11], inter-operator subjectivity in producing relative cerebral blood volume (rCBV) values, large variation in reported reliability of sequence processing and, disputed diagnostic criteria [6].

The inter-operator subjectivity can be explained by the often used ‘hot spot’ region of interest analysis, a lack of clinically validated cut-off values to differentiate TP from TRA, and a lack of standardization of imaging-acquisition techniques and postprocessing software [4, 10, 12–15]. More specifically, the ‘hot spot’ region of interest methodology is based on selecting and evaluating a region within the tumor with the highest perceived rCBV on a single image.

The’hot spot’ method, however, grossly underrepresents the complexity and heterogeneity of the region of interest (ROI) and substantially underestimates the volume of interest (VOI) as it often only partially covers the lesion in the most hyperintense region as seen on perfusion sequences. A more complete representation of the actual perfusion of the lesion can be acquired by segmenting its entire volume and then taking the mean rCBV of the entire contrast enhancing lesion. By selecting the entire volume, T1-weighted contrast-enhancing areas with low perfusion within the lesion are also taken into account and the heterogeneity in perfusion is thus accounted for in the mean rCBV value. The selection of the complete volume of the lesion allows for a mean rCBV value for the entire lesion and simultaneously allows for automatic detection of the actual highest rCBV value within a lesion (rCBVmax). Both of these metrics theoretically provide a more accurate representation of the perfusion of the lesion as a whole, compared to eyeballing the area of highest perfusion, which can be difficult in a lesion with multiple ‘hot spots’ or with mixed perfusion in the ROI. A more qualitative way of assessing perfusion data is a exclusively visual approach, in which the radiologist scores a tumor based on the visual perfusion without the use of rCBV values. Which of the mentioned techniques provides the best clinical differentiation between TP and TRA is not clear.

Therefore, the current study compared the diagnostic value of three approaches used to analyse DSC PW-MRI in distinguishing TRA from TP in post-treatment glioma patients. The compared approaches were I) a VOI based approach, II) a ‘hot spot’ based approach and III) a visual assessment of the same single-moment DSC PW-MRI data. Furthermore, the subjectivity of the ‘hot spot’ and visual assessment was studied. All analyses were carried out by independent experienced neuro-radiologists who were blinded to the outcome.

## Materials and methods

### Ethical approval, in- and exclusion criteria

Ethical approval was waived by our institution’s ethical review board due to the retrospective nature of this study. We evaluated patients with suspected tumor recurrence between January 2021 and May 2022. Inclusion criteria comprised: I) 18 years of age or older with histopathologically proven glioma, II) treated with surgical resection, chemotherapy and radiation therapy, III) enlarging or new contrast-enhancing mass on follow-up MRI, IV) available pre-contrast T_1_-weighted images, post-contrast T_1_-weighted images and DSC PW-MRI perfusion weighted images and V) histopathological examination after lesion biopsy or surgical resection or clinical and radiological follow-up to determine the ground truth of the new enhancing lesion (TRA vs. TP). Exclusion criteria included: I) non-contrast enhancing tumor on MRI, II) distortion of MR signal due to blood products or other artefacts, III) histopathological entities other than glioma and IV) patients that opted-out of participating to research. As the inclusion criteria require a new-enhancing mass to be visible on follow-up MRI the duration until the follow-up scan used in the analyses can differ.

### Defining the final outcome of the lesions

To determine if the final state of a lesion was TRA or TP, two methods were used in this study. If available, the gold-standard of histopathological confirmation was chosen as the primary means of determining the state of a lesion. If pathological material was unavailable, the often-used silver standard confirmation was used, consisting of clinical and radiological follow-up over a period of at least 3 months. The approach of clinical and radiological evaluation of the lesions over time complied with the RANO criteria [2].

### MRI acquisition protocol

All MR imaging was performed on a 1.5 Tesla scanner (Magnetom Avanto Fit, Siemens Healthineers, Erlangen). The protocol included a 3D T_1_-weighted MPRAGE sequence (repetition time (TR) 2100 ms; echo time (TE) 2.42 ms; Inversion time (Ti) 900 ms; isotropic voxel size 1 mm; acquisition time 5 min) before intravenous injection of gadoteric acid (Dotarem, Guerbet, Villepinte) as contrast agent. Then, a preload bolus of gadoteric acid contrast agent was administered by continuous flow of contrast (Dotarem 0.1 ml/kg bodyweight at a flow rate of 5 ml/sec). After this an axial multi-slice 2D T_2_-weighted sequence (TR 5310 ms; TE 85 ms; resolution 1 × 1x5 mm; acquisition time 2:30 min) and a 2D Fluid-Attenuated Inversion Recovery (FLAIR) sequence (2D, TR 9000 ms; TE 87 ms; inversion time 2500 ms; resolution 1 × 1x5 mm; acquisition time 3:30 min) was acquired. Then a bolus of contrast (20 ml Dotarem at 5 ml/sec) was injected, followed by a T_2_*-weighted sequence (TR 1350 ms; TE 43 ms; voxel size 1.8 × 1.8x5 mm;acquisition time 2 min) and a T_1_-weighted 3D fast turbo spin echo sequence (TR 600 ms; TE 7.1 ms; isotropic voxel size 1 mm; acquisition time 3:26 min) The susceptibility induced signal loss on T_2_*-weighted sequences, resulting from the passage of the contrast agent, was used to acquire the DSC PW-MRI data.

### Semi-quantitative ‘hot spot’ analysis

**Fig. 1:**
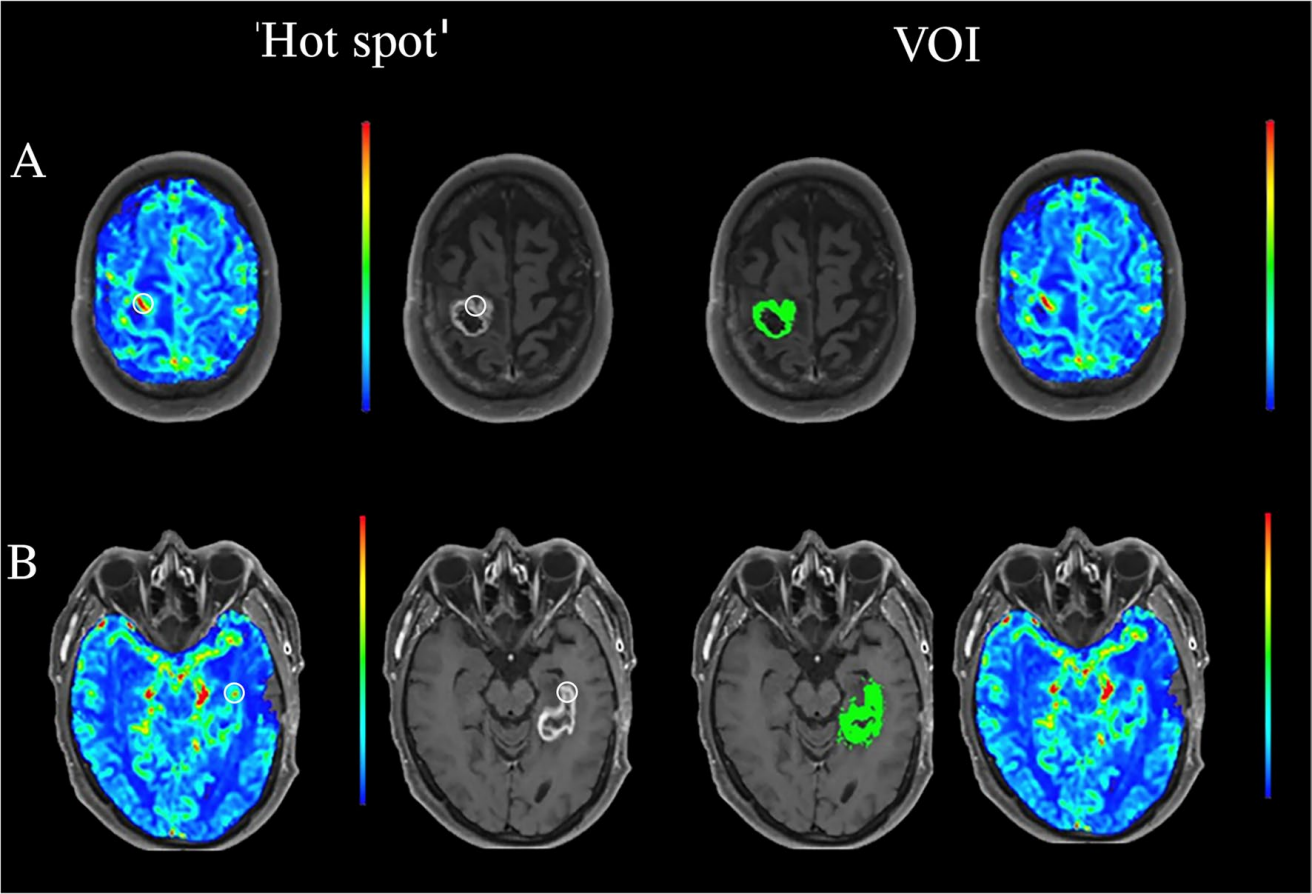
‘Hot spot’ and VOI processing of dynamic susceptibility contrast (DSC) images of two glioblastoma lesions. The white circular region of interest (ROI) markers seen in the DSC-PW (left) and post-contrast T1-weighted (right) sequences in the ‘hot spot’ column serve as an example of ROI placement using the ‘hot spot’ methodology commonly used in clinical practice. The area of slightly higher CBV measured in the perceived ‘hot spot’ correlates to a part of the enhancing region. In the VOI column the automatically selected lesion volume of interest in the same two patients can be seen in the post-contrast T1-weighted sequence (left), the respective lesion perfusion can be seen in the DSC-PW sequence (right). The contrast-enhancing region illustrated in A turned out to be tumor progression, while the lesions in B involuted over time and was therefore considered a treatment related abnormality

Three radiologists or radiology residents (between 5 and 23 years of experience in neuroimaging) were tasked with placing a 70 mm^2^ circular ROI on the perceived ‘hot spot’, as seen in Fig. 1. The readers were also asked to place a reference ROI of the same size in the contralateral centrum semiovale [16]. This reference placement has been shown to result in the most robust results when analysing perfusion metrics between different observers [17]. The mean rCBV value retrieved from the ROI placed on the ‘hot spot’ of the lesions was recorded as rCBV_hotspot_, while the reference was rCBV_ref_. The rCBV ratio was then calculated by dividing the rCBV_hotspot_ by the rCBV_ref_. This was done to take the variation in base hemodynamics between patients into account, making the produced rCBV ratio a more standardized measure of perfusion.

### Semi-automatic VOI analysis

A workstation equipped with OsiriX MD (Version 12.0; http://www.osirix-viewer.com) and a commercially available plug-in (IB Neuro; Imaging Biometrics, Elm Grove, Wisconsin), which uses a leakage-correction algorithm to process perfusion data and calculate perfusion maps [15, 18, 19] was used for this analysis. For semiautomated image analysis, we used IB Rad Tech (Imaging Biometrics, Elm Grove, Wisconsin), which is a workflow engine that plots rCBV maps in semi-automatically defined volumes of interest [18, 20, 21]. A standardisation of the cerebral blood volume map was automatically performed by IB Rad Tech using a standardisation protocol developed by Bedekar et al. [22]. To visualise the T1-enhancing lesion more clearly IB Rad Tech first subtracted the intensities of the standardized pre-contrast T1 from the standardized post-contrast T1, creating a ΔT1-weighted sequence. This was followed by the semi-automatic selection of defined volumes of interest, which concerns the manual drawing of an ROI around the ΔT1-weighted area of interest, after which the protocol automatically selected the voxels that showed contrast enhancement in the drawn ROI, thus creating a complete selection of the volume of interest as seen in Fig. 1. Mean and maximum rCBV values and volumetric parameters of the contrast enhancing VOIs were automatically generated by the software package for each lesion. This semi-automatic analysis was done by a single radiology resident (D.H.) with profound experience using the software. This approach was chosen because the standardization protocol of the software package is known to produce highly reproducible rCBV values as it does not rely on manual reference placement and compensates for variability in acquisition differences [22–24]. Secondly, the semi-automatic VOI selection based on contrast-enhancing pixels in the ΔT1-weighted sequence left little room for inter-operator variability, as it is only susceptible to other contrast enhancing artifacts such as blood vessels, which trained radiologists should be able to identify.

### Visual assessment of DSC PW-MRI data

Three radiologists or radiology residents with between 5 and 23 years of experience in (experimental) neuroimaging assessed the DSC PW-MRI data visually or by drawing an ROI and recorded their suspected outcome of the lesion, being either TP or TRA. These predictions were mainly based on the perfusion of the lesion, as it is known that features observed on conventional imaging do not contribute to accurate differentiation [3]. This analysis was based on a single imaging session, so there was no follow-up or predating imaging available during the analysis. The analysis was performed on a workstation equipped with Syngo.via VB60 (Siemens Healthineers, Erlangen, Germany). Next to the DSC PW-MRI sequences, the readers were provided with a T_1_-weighted sequence pre- and post-contrast and a T_2_-FLAIR sequence to aid with anatomical orientation and lesion identification. The colour scale used to analyse the PW-MRI data was spectrum 10-step, a calibrated colour scale provided in the Syngo.via program, meaning that the colours visible were chosen to fit on a set scale, meaning that the impact of the chosen colours on decision making is minimized. The predicted outcome was then compared to the definitive outcome. The definitive outcome was determined based on available clinical and radiological follow-up and/or histopathological data. The definitive outcomes were then dichotomized by one of the authors, who was still blinded to the definite outcome at this stage of the research process.

### Statistical analyses

IBM SPSS Statistics for Windows (version 28.01.1, Armonk, USA) was used for most of the statical analyses of this study. To assess the variability between the different readers of the ‘hot spot’ DSC PW-MRI data the intraclass correlation coefficient (ICC) was determined for the measured rCBV ratio, rCBV_hotspot_ and rCBV_ref_. After this, the ICC was used to assess the inter-observer variability of the rCBV ratio measured in the ‘hot spot’ analysis.

Normality of the complete VOI segmentation voxel-wise rCBV data was assessed by use of the Kolmogorov–Smirnov test. When the data was normally distributed, mean, and standard deviation values were compared between groups using the independent Student’s t-test. If the data showed a non-normal distribution, the Mann–Whitney U-test was used to compare the median and range data between patients with TRA and TP. To evaluate whether the volume of the lesion had any correlation with the rCBV values of the lesions, a Pearson Correlation analysis was carried out.

The visual assessment data was analysed using contingency tables, resulting in the sensitivity and specificity achieved per reader. Cohen’s kappa was then used as a measure for the interobserver agreement.

A Receiver Operating Characteristic (ROC) curve was created to determine a VOI-based rCBV cut-off value with a balanced sensitivity–specificity ratio, ultimately able to classify TRA and TP. The same analysis was done to determine the semi-quantitative ‘hot spot’ based rCBV ratio cut-off value, able to classify TRA and TP. In both cases the most balanced sensitivity–specificity ratio was determined by selecting the point on the ROC-curve most closely located to the top-left corner of the unit square. The corresponding rCBV cut-off value of this sensitivity–specificity ratio was then retrieved from SPSS, together with an AUROC value indicating the diagnostic accuracy. Then Delong’s test as available in the pROC package for R [25] was used to compare the AUC’s of the created ROC curves, for this analysis R was used (version 4.3.1, R Foundation for Statistical Computing, Vienna, Austria.).

## Results

### Demographics of the included patients

In total, 50 glioma patients were included in this study. Based on the WHO classification of gliomas [26], thirty-eight patients had a medical history of glioblastoma (76%), nine patients were treated for an IDH-mutant astrocytoma grade 2–4 (16%) and three patients were previously diagnosed with an oligodendroglioma (8%). An overview of the included patients can be found in Table 1.

**Table 1:** Characteristics of the included post-treatment glioma patients

Patient characteristics	TR (*n* = 29)	TRA (*n* = 21)
Mean age	53.1 ± 11.7 years	60.4 ± 12.8 years
Sex	15 male; 14 female	12 male; 9 female
Tumor type and WHO grade	Astrocytoma WHO 2 (2), Astrocytoma WHO 3 (1), Oligodendroglioma WHO 3 (2), Glioblastoma WHO 4 (24)	Astrocytoma WHO 2 (2), Astrocytoma WHO 3 (2), Astrocytoma WHO 4 (1), Oligodendroglioma WHO 2 (1), Oligodendroglioma WHO 3 (1), Glioblastoma WHO 4 (14)
IDH-mutation status	mutation (6), wildtype (21), unknown (2)	mutation (7), wildtype (14)
MGMT-promotor methylation status	methylation (13), no hypermethylation (7), unknown (9)	hypermethylation (14), no-hypermethylation (1), unknown (6)
1p/19q status	co-deletion (2) no deletion (7) partial deletion (1) unknown (19)	co-deletion (2), no deletion (8), unknown (11)
Median volume of total lesion	54.6 mL (1.3–596 mL)	15.6 mL (1.2–187 mL)

Three patients underwent stereotactic biopsy; in those cases, histopathological confirmation of the tumor outcome was used. In all other included patients, the final outcome was defined by use of clinical and radiological follow-up over a follow-up period of at least three months. In twenty-nine cases, the new contrast enhancing lesion was found to reflect TP (58%). In the other twenty-one cases the enhancing region turned out to involute over time and was interpreted as TRA. The mean ages in the TP and TRA groups differed significantly (t(48) = 2.09, *p* = 0.04).

### Statistical analysis of the semi-quantitative ‘hot spot’ data

The ICC of the measured rCBV ratio was 0.83 (95%-CI: 0.73–0.90). While the ICC of the measured rCBV_hotspot_ was 0.89 (95%-CI: 0.82–0.93) and the ICC of the rCBV_ref_ was 0.54 (95%-CI: 0.26–0.72). The ICC of a comparison between the ‘hot spot’ rCBV ratio and the rCBV_mean_ from the VOI assessment, resulted in an ICC of 0.72 (95%-CI: 0.56–0.83).

### Statistical analysis of the VOI PWI data

The Kolmogorov–Smirnov test showed that the VOI rCBV_mean_ data was normally distributed (D(50) = 0.09, *p* = 0.20), whereas the rCBV_max_ and volumetric data showed a non-normal, right-skewed distribution (D(50) = 0.13, *p* = 0.03 and D(50) = 0.28, *p* < 0.001 respectively). The retrieved rCBV values and volumes per group can be seen in Table 2.

**Table 2:** rCBV values of the TR and TRA group based on the Volume of Interest-analysis

	TR	TRA
Mean rCBV_mean_	1.5 mL/100 g (SD ± 0.6 mL/100 g)	0.8 mL/100 g (SD ± 0.5 mL/100 g)
Median rCBV_max_	7.6 mL/100 g (SD ± 4.1 mL/100 g)	3.4 mL/100 g (SD ± 3.2 mL/100 g)
Median volume	54.7 ml (ranging from 2.0–60 ml)	15.6 ml (ranging from 0.12–18.9 ml)

Significant differences were observed between the measured rCBV_mean_ and rCBV_max_ values in TP and TRA lesions (t(48) = 4.3, *p* < 0.001 and U = 168, *p* = 0.007 respectively). The median volumes in the TP and TRA lesions also showed significant differences between the groups (U = 175, *p* = 0.011). Pearson correlation analysis showed that rCBV_max_ value was significantly correlated with volume of the lesions (*p* < 0.001; r = 0.64). The rCBV_mean_ value, on the other hand, was not significantly correlated with volume of the lesions (*p* = 0.24; r = 0.17).

### Statistical analysis of the visual assessment data

The radiologists or radiology residents were anonymized as reader X, Y and Z, their results can be seen in Table 3. The inter-observer agreement between reader X and Y was κ = 0.65, between reader X and Z it was κ = -0.72 and between reader Y and Z it was κ = -0.98.

**Table 3:** Analysis of the correctness per researcher during the visual assessment

Reader X	TP	TRA
Predicted TP	12	2
Predicted TRA	17	19
	Sens = 41%	Spec = 91%
Reader Y	TP	TRA
Predicted TP	15	3
Predicted TRA	14	18
	Sens = 52%	Spec = 86%
Reader Z	TP	TRA
Predicted TP	25	14
Predicted TRA	4	7
	Sens = 86.2%	Spec = 33%

Sensitivity being the percentage chance of identifying TR, while specificity is the percentage chance of identifying TRA

### Diagnostic accuracy of different post-processing methods of PWI data

**Fig. 2:**
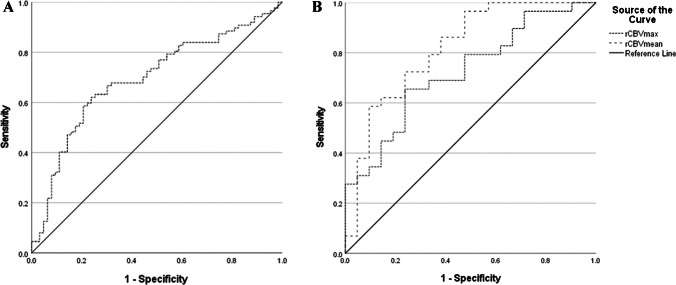
Receiver operator characteristics (ROC) curves based on the ‘hot spot’ (**A**) and semi-automatic volume of interest (**B**) analysis of relative cerebral blood volume (rCBV) data to distinguish tumor progression from treatment related abnormalities. Area under the ROC curve (AUROC) for the rCBV ratio of the ‘hot spot’ analysis was 0.69 (95%-CI: 0.61–0.78). The AUROC of rCBV_max_ and rCBV_mean_ are 0.72 (95%-CI: 0.58–0.86) and 0.82 (95%-CI: 0.70–0.94) respectively

The Receiver Operating Characteristic (ROC) curves can be found in Fig. 2; in both curves TP was seen as the positive state. The VOI-based analysis showed an AUROC of 0.72 (95%-CI: 0.58–0.86) and 0.82 (95%-CI: 0.70–0.94) for rCBV_max_ values and rCBV_mean_ values, respectively. Regarding rCBV_max_, a cut-off value of 5.1 mL/100 g resulted in a sensitivity of 69% and specificity of 67%. A cut-off value of the rCBV_mean_ of 1.11 mL/100 g resulted in a balanced sensitivity and specificity value of 72% and 76%, respectively, to distinguish TP from TRA.

For the semi-quantitative ‘hot spot’ analysis an ROC curve based on measured rCBV ratio was made based on the combined analysis of the three readers. This resulted in an AUROC of 0.69 (95%-CI: 0.61–0.78). The achieved sensitivity and specificity of this analysis is 67% and 70%, based on a balanced rCBV cut-off of 1.63 mL/100 g. DeLong’s test used on the semi-automatic VOI analysis ROC curve indicated a significant difference between the AUROC of the rCBV_mean_ and rCBV ratio from the ‘hot spot’ analysis (Z = -2.26, *p* = 0.023). It also showed a significant difference between the AUROC of rCBV_mean_ and rCBV_max_ (Z = -2.655, *p* = 0.0079) and a non-significant difference between the AUROC of the rCBV_max_ and the rCBV ratio from the ‘hot spot’ analysis (Z = -0.50, *p* = 0.61).

## Discussion

This study illustrates that at its current state the usage of VOI based DSC PW-MRI analysis compared to a ‘hot spot’ or purely visual approach results in a slightly higher diagnostic accuracy. It also elucidates the subjectivity of the ‘hot spot’ and visual approach, which is mostly circumvented by the usage of VOI based analyses.

When used to distinguish TP from TRA in this study, a VOI cut-off value of rCBV_mean_ of 1.11 mL/100 g resulted in a sensitivity and specificity value of 72% and 76%, respectively (AUROC = 0.82). In comparison, the highest scoring observer using the visual assessment methodology, had a sensitivity and specificity of 41% and 91% respectively. The sensitivity and specificity achieved with ‘hot spot’ assessment of DSC perfusion data was 67% and 70% respectively (AUROC = 0.69). While the achieved sensitivity and specificity in this study are mediocre, it should be noted that in clinical reality a treatment decision would be made after a follow-up period and with availability of predating scans, in that setting a higher sensitivity and specificity is to be expected. This study is meant to analyse the difference in predictive accuracy of these different techniques based on a single moment analysis.

On a group level, the rCBV_max_ of a lesion showed a moderate correlation with the volume of the lesion (*p* < 0.001; r = 0.64), while the mean rCBV value does not seem to be influenced by the volume (*p* = 0.24; r = 0.17). The nature of this correlation on an individual level has not been tested in this study, so the assumption that a large lesion will have a higher rCBV_max_ and is thus more likely to be TP should not be made without consulting other means of differentiating TP and TRA. The differences found in rCBV_max,_rCBV_mean_ and median volumes between the TP and TRA group in this study match the hypothesis that perfusion is different between the two mentioned states. However, as the ages between the groups differ significantly (t(48) = 2.09, *p* = 0.04), with TRA having a higher mean age, this comparison should be used with caution as the age difference could also potentially explain the found differences.

DSC PW-MRI in the radiological follow-up of post-treatment glioma lesions is widely used to distinguish TRA from TP. Three recent reviews on this topic provided a sensitivity ranging from 83–93% and a specificity ranging from 75–88%, indicating good diagnostic accuracy with regard to distinguishing TRA from TP [8–10]. However, the included studies in these three meta-analyses mainly used the ‘hot spot’ technique. Next to that these studies differed from ours, as some of the studies they base their results on differentiated between pseudoprogression and radiation necrosis, while we used TRA as a term to cover both. Included studies also often used predating or follow up scans in their analysis and not single moment data. In this study the single-moment ‘hot spot’ methodology was performed by three experienced neuro-radiologists and resulted in an AUROC (0.69) which was significantly lower than the AUROC of the ROC graph based on the rCBV_mean_ from the VOI study (0.82), as tested with the DeLong’s test (Z = -2.26, *p* = 0.023). In the ‘hot spot’ assessment, the ICC showed poor reliability in the placement of references (ICC = 0.54), despite using a clearly communicated reference placement in the contralateral centrum semiovale that should have had the least inter-observer variability [17]. This might be explained by variation in the chosen slice in which the readers chose to place their reference, however this level of subjectivity with an agreed placement of reference underlines the weakness of manual placement. The placement of the ‘hot spot’ itself resulted in an excellent reliability (ICC = 0.89) between the observers, indicating that the manually placed ‘hot spot’ does provide similar values between researchers. Besides that, the ‘hot spot’ placement also showed moderate reliability when compared to a complete VOI analysis (ICC = 0.72), indicating that if done correctly the ‘hot spot’ placement does match the rCBV found in a complete VOI analysis.

Additionally, a single moment visual assessment of the same DSC perfusion data was also included in this study, showing large differences between the achieved sensitivity and specificity per neuro-radiologist, ranging from 41–86% and 33–91% respectively. The found agreement between the neuro-radiologists or residents differed from poor (κ = -0.98, κ = -0.72) to substantial (κ = 0.65), indicating that there was a high degree of subjectivity. While this assessment deviated from clinical practice due to a lack of available follow-up or predating images, the found sensitivity–specificity ratios found per radiologist indicate that in order to get a high sensitivity or specificity they greatly sacrifice the other. In this diagnostic dilemma a high specificity is preferred, as you want to correctly diagnose TP to allow for earlier treatment, but the sacrificed sensitivity means that TP is often missed if specificity is prioritized.

A comparison between the inter-operator agreement of the ‘hot spot’ analysis and the agreement between the readers in the visual assessment illustrates that the visual approach is most susceptible to subjectivity. In the visual assessment the differences between readers were very large, while in the ‘hot spot’ approach the placement of the ROI was reliable between all readers. Only the reference placement showed variability. We feel the disagreement in the visual assessment reflects the clinical tendencies of radiologists as the expert readers were not instructed to act more defensively or otherwise. In this case a defensive choice would be to opt for the ‘worst-case’ scenario, being TP. Reader Z chose to utilise a more defensive approach than the others, resulting in more frequent TP diagnoses (predictions reader Z: 39 TP, reader X: 14 TP and reader Y: 18 TP), thus causing the found disagreement. This disagreement underlines the subjectivity of visual assessment even between trained radiologists.

All in all, this study confirms that a single-moment exclusively visual assessment of post-operative glioma DSC PW-MRI data is vulnerable to inter-observer variability. This finding and the low ICC found in the reference placement of the ‘hot spot’ analysis matches the high inter-observer variability found in Kouwenberg et al. [27] and Smits et al. [16]. Both studies describe that the placement of ‘hot spots’ and references in post-treatment glioma DSC PW-MRI data shows low reliability and reproducibility. It is therefore recommended that if rCBV is visually measured in post-operative glioma patients it should be carried out by two readers and with precaution.

The use of semi-automatic complete VOI DSC has been attributed good discriminative power to distinguish TP from TRA. For example, in a recent study rCBV values of a complete VOI obtained from DSC PW-MRI yielded an AUROC of 0.81 to distinguish TRA from TP [21]. Similar results were obtained in the rCBV values of a complete VOI analysis of DSC PW-MRI data in the setting of metastatic neuro-oncological disease. In the study of Kuo et al., thirty subjects with 37 lesions were investigated (20 TRA; 17 TP). When using rCBV values of a VOI obtained from DSC PW-MRI data, an AUROC of 0.79 was yielded to discriminate TRA from TP [20]. The outcome of these papers is corroborated by the current results, as we found an AUROC of 0.82 (95%-CI: 0.70–0.94) for the mean rCBV in the semi-automatic VOI analysis.

It has been reported that implementation of DSC PW-MRI in routine follow-up MRI of glioma can aid the early diagnosis of TP [28]. In this study, a standardised perfusion acquisition protocol and standardised methodology to process data with well-validated criteria was used. This has been recommended by others for research on post-treatment radiological evaluation of glioma patients [13]. However, harmonization of the imaging protocol and the post-processing work-flow remains lacking, probably explaining the wide range of cut-off values that have been reported in literature [6]. Also, these differences hinder the sharing and pooling of imaging data. Nevertheless, important steps with regard to the standardisation of DSC acquisition parameters have been taken during the last years [12–14].

### Strengths and limitations

In the VOI analysis, the entire T1-weighted contrast enhancing lesion was included, which limits the inter- and intra-observer variability when compared to manual placement of regions of interest. The used IB-Rad Tech software and it’s semi-automatic processing of DSC-PWI images has been described to further reduce user-related variability [22], as its automated standardisation circumvents the manual placement of references which was shown to be especially vulnerable to subjectivity. Another strength of the current study concerns the fact that for the first time, single-moment rCBV values of VOIs were directly compared with rCBV values of ‘hot spots’ and a purely visual assessment, which are commonly used in standard clinical reading of DSC PW-MRI data in the post-treatment evaluation of gliomas to distinguish TP from TRA.

An important limitation of the current study comprised its retrospective nature and the lack of an external validation cohort in which the observed threshold values can be tested. It must be emphasized that the differences between the rCBV values of the VOIs were valid on a group level; the usefulness of this technique in individual patients needs further investigation.

The single-moment analysis of the PWI data can also be seen as a limitation, as a three to six-month period of follow-up imaging and pre-operative scans are usually utilised to differentiate between TP and TRA. However, readers were informed with regard to the fact that the MR data they read were the first imaging study on which a new or growing contrast-enhancing lesion appeared. Therefore, it was not expected that this single-moment analysis impacted the outcome of the reader study.

The heterogeneity of the studied population can be seen as a limitation as they possess inherent differences in malignancy grade and therefore in behaviour and treatment options. However, all included subtypes can progress on follow-up imaging and can show new enhancing lesions in the post-treatment setting. As this study investigated the diagnostic accuracy of three approaches to analyse DSC PW-MRI data when new contrast-enhancing lesions occur, the impact of the rather heterogenous population is considered minimal.

The small number of patients per subgroup (when stratified by for example tumor type or treatment schedule) prevented a statistically sound analysis of the used subgroups and identification of potential differences between the used pathologies. As the scope of this study was aimed at elucidating differences between the used methodologies, this was deemed acceptable by the authors, however a future study with larger subgroups per pathology and treatment used should aid in the identification of potential differences.

A final limitation is the usage of a 1,5 T MRI system instead of a 3 T system. A 3 T scanner would allow for an increased signal-to-noise (SNR) ratio, increased temporal- and spatial resolution. However, DSC PW-MRI is often not limited by SNR, and the usage of 1,5 T in glioma imaging shows an almost perfect correlation with 3 T in MR modalities such as rCBV and identified lesion volume [29] so using a 1,5 T system should be sufficient in answering our study objectives.

## Conclusion

This paper shows that a semi-automatic processing of the complete lesion achieves a greater diagnostic accuracy in the post-treatment radiological follow-up of glioma patients on a group level compared to a ‘hot spot’ or visual approach in a single moment analysis of DSC PWI data. The visual and ‘hot spot’ based approaches are also more subject to inter-observer variability, especially in the placement of references.

## Data Availability

The datasets generated during and/or analyzed during the current study are not available to ensure complete patient anonymity.

## Abbreviation

ASL: Arterial Spin Labelling
AUROC: Area Under the Receiver Operating Characteristics
DCE: Dynamic Contrast Enhancement
DSC: Dynamic Susceptibility imaging
FLAIR: Fluid Attenuated Inversion Recovery
ICC: Intraclass correlation coefficient
IDH: Isocitrate Dehydrogenases
MGMT: Methylguanine Methyltransferase
PWI: Perfusion Weighted Imaging
RANO: Response Assessment in Neuro-Oncology
rCBV: Relative Cerebral Blood Volume
ROC: Receiver Operating Characteristic
ROI: Region Of Interest
TE: Echo Time
Ti: Inversion time
TP: Tumor Progression
TR: Repetition Time
TRA: Tumor Related Abnormalities
SNR: Signal-to-noise ratio
VOI: Volume Of Interest
WHO: World Health Organization
